# Combination chemotherapy with Regorafenib in metastatic colorectal cancer treatment: A single center, retrospective study

**DOI:** 10.1371/journal.pone.0190497

**Published:** 2018-01-05

**Authors:** Chun-Yu Lin, Tseng-Hsi Lin, Chou-Chen Chen, Ming-Cheng Chen, Chou-Pin Chen

**Affiliations:** 1 Division of Colorectal Surgery, Department of Surgery, Taichung Veterans General Hospital, Taichung, Taichung, Taiwan; 2 Division of Hematology, Department of Internal Medicine, Taichung Veterans General Hospital, Taichung, Taiwan; Universidade do Algarve Departamento de Ciencias Biomedicas e Medicina, PORTUGAL

## Abstract

**Background:**

Regorafenib has been demonstrated as effective in refractory metastatic colorectal cancer. Combination use with chemotherapy has not been reported. We examined the efficacy and safety of adding chemotherapy to Regorafenib for the treatment of metastatic colorectal cancer(mCRC) patients.

**Methods:**

We recruited mCRC patients at our institute who received either regorafenib monotherapy or regorafenib in combination with other chemotherapies. All patients had received chemo and target therapies and presented with disease progression before regorafenib treatment. The primary end point was overall survival.

**Findings:**

Between September1, 2015 and May 31, 2017, 100 mCRC patients at our institute received regorafenib treatment. 39 patients were excluded due to poor performance, lack of timely treatment, or inadequate clinical data. A total of 34 patients received regorafenib combined with other chemotherapies, and 27 patients received regorafenib alone. Median follow up time was 10.4 and 6.1 months, respectively. The primary end point of median OS was higher in the combination group than in the single use group (20.9m vs 10.3m, *p* = 0.015). The most frequent adverse events were hand-foot skin reactions(16[47.1%]vs 12[44.4%]), fatigue(6[17.6%] vs 7[25.9%]), gastrointestinal discomfort (7[20.6%] vs 6[22.2%]), neutropenia (4[11.8%] vs 1[3.7%]), diarrhea(4[11.8%] vs 1[3.7%]), and mucositis(5[14.7%] vs 1[3.7%]).

**Conclusion:**

The present study showed the efficacy and side effects of regorafenib combination treatment. Superiority in median OS and median PFS was noted in the combination group. The sampling difference between the study and observation groups effects justifies the comparison. Further clinical evidence of combination therapy efficacy is pending future studies.

## Introduction

During the past few decades, the incidence of colorectal cancer has increased worldwide[1].There were 1,363,000 newly diagnosed cases worldwide, which led to 693,900 deaths in 2012[2,3]. The colorectal incidence in Taiwan(44.32/100000) is high. There were 15,140 newly diagnosed cases leading to 5,265 deaths in 2013[4]. Approximately 25% of patients with colorectal cancer have metastatic disease with a clinically significant detrimental effect on prognosis[5,6]. With the administration of chemotherapy and target therapy, the median overall survival time of metastatic colorectal cancer(mCRC) has improved from 12 to 33 months(in KRAS wild-type patients)[7,8].However, the response rate of chemotherapy apparently decreased in the further therapies[9–12]. The identification of a chemotherapy with a higher response rate has attracted increasing attention and is addressed in the present study.

Regorafenib is an orally available, small-molecule multikinase inhibitor that targets signaling pathways implicated in tumor angiogenesis(VEGF receptors 1–3 and TIE2), oncogenesis(KIT, RET, RAF1, and BRAF), and the tumor microenvironment (platelet-derived growth factor receptor and fibroblast growth factor receptor)[12]. Evidence of activity of regorafenib in metastatic colorectal cancer was demonstrated in two international randomized Phase III trials(CORRECT and CONCUR)[13,14]. These trials indicated that regorafenib monotherapy improved overall survival compared with the placebo dose (6.4 months vs 5.0 months, hazard ratio[HR] 0.77, 95% CI 0.64–0.94; *p* = 0.0052 in CORRECT and 8.8 months vs 6.3 months, [HR] 0.55, 95% CI 0.40–0.77; *p* = 0.00016 in CONCUR). However, adverse events (AE) of regorafenib were apparent, which were most likely observed as hand-foot skin reaction(HFS), diarrhea, fatigue, and elevated liver function. Adverse events are more frequent observed in Asians, and tolerance was lower[15].Currently, clinical practice use a dose-escalation protocol at certain institutes[16,17].which may increase drug compliance and with relatively the same effect.

Regorafenib was approved for the treatment of mCRC by the US Food and Drug administration in September 2012[18], and approved in mCRC treatment by the Taiwan National Health Insurance Scheme (NHI) since September 2015[19]. The superiority of combination use of target therapy with chemotherapy was demonstrated in Cetuximab in 2004[20]. The effect of regorafenib combination use remains unknown. We collected mCRC patients from our institute who had received regorafenib combined with chemotherapy and compared these individuals with patients who received only reforafenib to observe differences in the therapeutic effects and side effects between these groups.

## Methods

### Study design and patients

**Fig 1:**
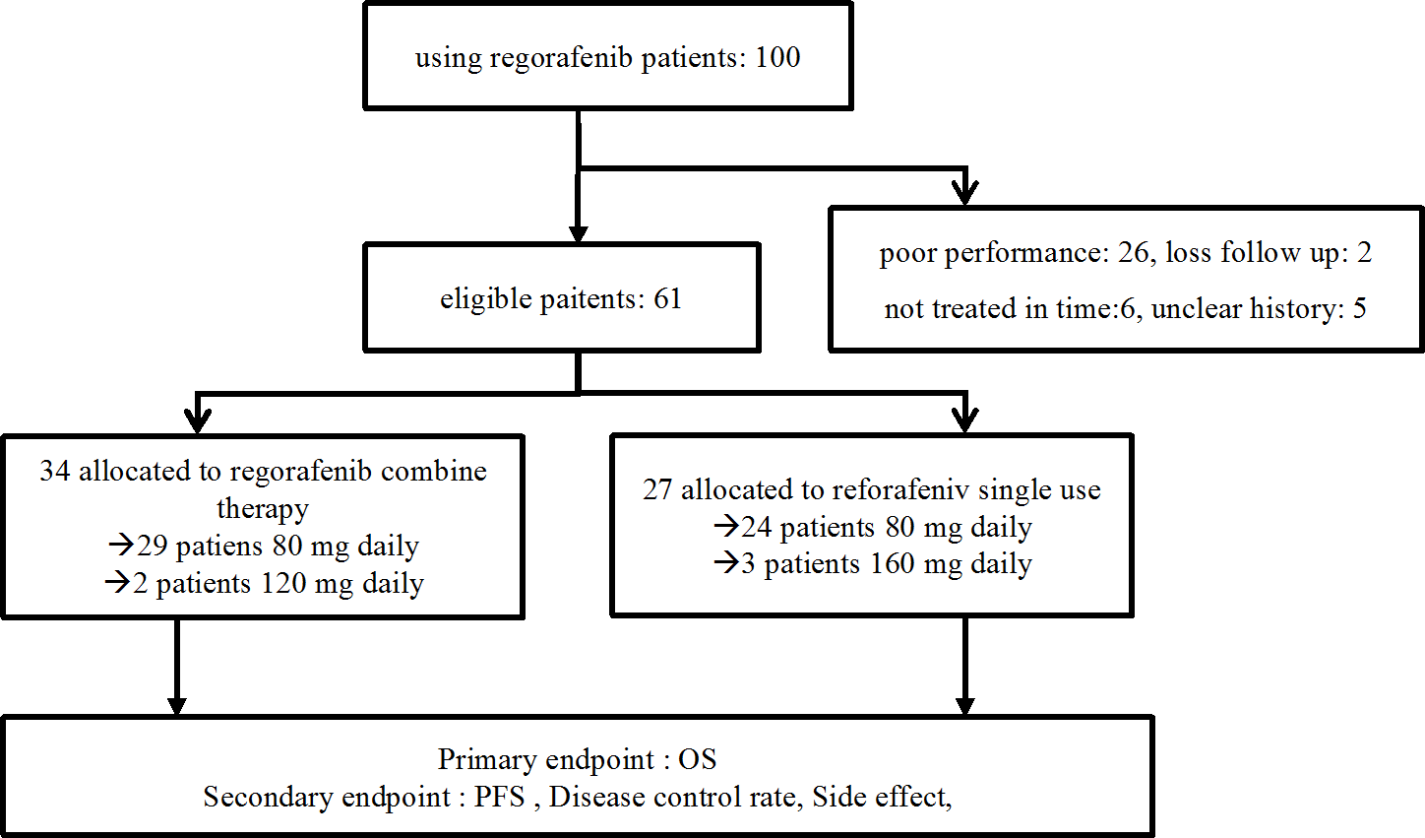
The profile of the present study.

**Fig 2:**
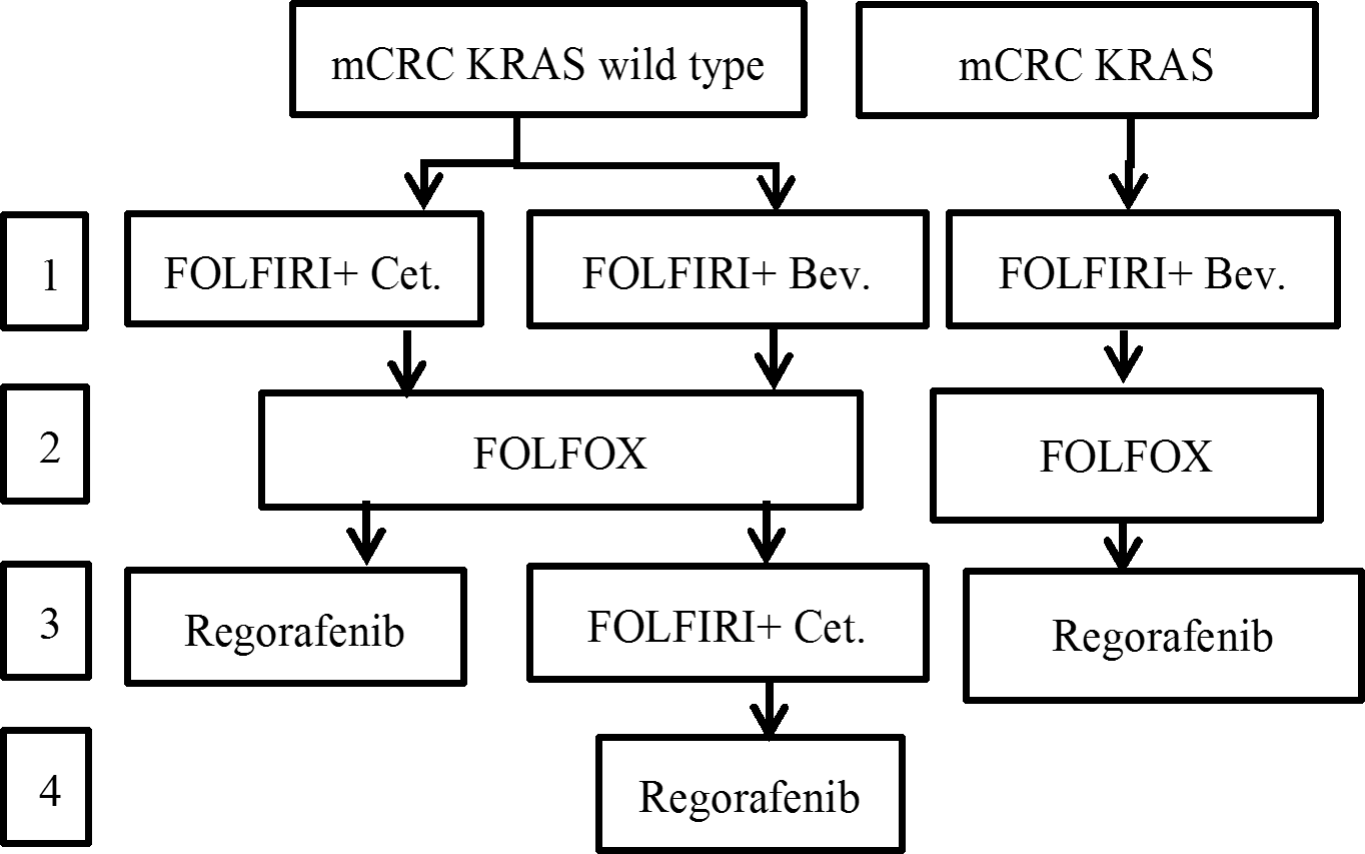
Taiwan National Health Insurance payment guidelines for metastatic colorectal cancer in September 2015.

The present study was a retrospective cohort study; data was collected during September 2015 to May 2017 in a single institute (Fig 1). In Taiwan, chemotherapy for metastatic colorectal cancer was paid for by the NHI according to NCCN guidelines[21]. Irinotecan-based chemotherapy (FOLFIRI), and anti-VEGF or anti-EGFR(for k-ras wild type) target therapy were the first line chemotherapies. If the disease progressed, then the patients received a second-line oxaliplatin based(FOLFOX) chemotherapy. In the third-line chemotherapy, anti-EGFR target therapy was used for KRAS wild type, and if anti-VEGF was used in first line, then regorafenib was administered to (Fig 2).

Eligible patients in the present study were pathologically confirmed for colorectal cancer, presented with metastasis and had received target therapy with disease progression within 3 months. The evaluation scale for measurable or non-measurable metastatic disease is according to Response Evaluation Criteria in Solid Tumor(RECIST) version 1.1.23[22]. The patients were adults (>18 years of age) with an Eastern Cooperative Oncology Group performance status of 0 or 1 and adequate bone-marrow, liver, and renal function at the start of the treatment.

### Procedure

We collected data from patients who received regorafenib therapy for metastatic colorectal cancer from September 2015 to May 2017. Patients with an expected life span of fewer than 3 months, brain metastasis, double cancer, regorafenib treatment further than fourth line therapy, or an unclear therapy record were excluded. We verified that the patient records were de-identified and anonymous.

Because of the adverse events of regorafenib, the standard dose was not applicable in every patient. Most physicians applied a dose-escalation protocol, which started with regorafenib at 80 mg per day with possible dosage modifications on each OPD to manage drug concentration and toxic effects. In the study group, there were chemotherapy agents used with regorafenib therapy of either 5-FU based agents, oxaliplatin, irinotecan, FOLFOX, or FOLFIRI. The choice of chemotherapy was determined according to the physicians' discretion. The dosage of these drugs was determined according to previous study. Tegafur /Uracil: 300 mg/m^2^/day, capecitabine: 2500 mg/m^2^, oxaliplatin: 85 mg/m^2^ per 2 weeks, irinotecan: 180 mg/m^2^ per 2 weeks, modified FOLFOX6: 2 hour infusion of oxaliplatin (85mg/m^2^) and folinic acid (100 mg/m^2^) followed by a 46 hours of continuous infusion of 5-FU (2,400 mg/m^2^), FOLFIRI: 90 minutes infusion of irinotecan (180 mg/m^2^) and folinic acid (100 mg/m^2^) followed by a 46 hours of continuous infusion of 5-FU (2,400 mg/m^2^). In the control group, regorafenib was used without any chemotherapy. Clinical condition was followed in the outpatient department(OPD) or during hospitalization every 2 weeks, including history-taking, a physical exam, and a laboratory exam. The tumor marker, CEA and CA-199, were assessed every 6–8 weeks, and chest X-ray, abdomen CT, or PET were performed every 10–14 weeks or once if there was a clinical condition that needed to be performed. Survival status was collected by a colorectal cancer case manager in the hospital (Table 1).

**Table 1:** Patients characteristic in the present study.

	Single use (n = 27)		Combine (n = 34)		*p* value
	n	%	n	%	
**Sex**					1.000
F	12	(44.4%)	14	(41.2%)	
M	15	(55.6%)	20	(58.8%)	
**Age**†	60.0	(54.0, 73.0)	63.0	(58.8, 73.3)	0.292
**ECOG**					0.162
0	2	(7.4%)	8	(23.5%)	
1	25	(92.6%)	26	(76.5%)	
**Drug**					
XeLIRI or FOLFIRI	0	(0.0%)	5	(14.7%)	
Ufur or Xeloda	0	(0.0%)	7	(20.6%)	
FOLFOX	0	(0.0%)	1	(2.9%)	
Irino	0	(0.0%)	19	(55.9%)	
Oxa	0	(0.0%)	2	(5.9%)	
**Initial dosage (mg)**					0.429
80	24	(88.9%)	29	(85.3%)	
120	0	(0.0%)	2	(5.9%)	
160	3	(11.1%)	3	(8.8%)	
**Tumor site**					0.039*
Cecum	3	(11.1%)	0	(0.0%)	
Ascending colon	1	(3.7%)	3	(8.8%)	
Transverse colon	1	(3.7%)	0	(0.0%)	
Descending colon	0	(0.0%)	5	(14.7%)	
Sigmoid colon	5	(18.5%)	11	(32.4%)	
Rectum	17	(63.0%)	15	(44.1%)	
**Side**					0.447
Right	5	(18.5%)	3	(8.8%)	
Left	22	(81.5%)	31	(91.2%)	
**Ras mutation**					1.000
Wild type	12	(44.4%)	15	(44.1%)	
Mutant type	15	(55.6%)	19	(55.9%)	
**Time when regorafenib was used**					0.940
2nd line	8	(29.6%)	10	(29.4%)	
3rd line	14	(51.9%)	18	(52.9%)	
4th line	5	(18.5%)	6	(17.6%)	
**Had received adjuvant therapy in stage 3**					
FOLFOX	8	(29.6%)	11	(32.4%)	1.000
**Target therapy beforeregorafenib**.					0.153
Bevacizumab	17	(63.0%)	20	(58.8%)	
Cetuximab	6	(22.2%)	3	(8.8%)	
Bevacizumab+ Cetuximab	4	(14.8%)	11	(32.4%)	
**Chemotherapy before regorafenib**					1.000
FOLFIRI /XeLIRI	12	(44.4%)	16	(47.1%)	
FOLFOX	0	(0.0%)	0	(0.0%)	
FOLFIRI and FOLFOX	15	(55.6%)	18	(52.9%)	

Chi-square test.

†Mann-Whitney U test, Median (IQR)

*p<0.05

**p<0.01

### Ethics statement

The present study was approved by ethics committees of Taichung Veterans General Hospital in Institutional Review Board(II) 106-B-06 Board Meeting. IRB number: CE17123B. The patients provided informed written consent to have data from their medical records used in the present study. We verified that patient records were de-identified and anonymously analyzed.

### Outcome

The primary endpoint was overall survival(OS) (time from regorafenib initiation to mortality by the disease). For these patients, regorafenib might have been the last line treatment. Median overall survival time reflected the disease control rate of the drug. Secondary endpoints were progression-free survival(PFS) (time from regorafenib initiation to clinical evidence including symptoms or radiologic images of disease progression), the proportion of patients who achieved disease control 3 months after treatment, and adverse effects (any grade of symptomatic event, and hematological events). We reported adverse events and laboratory abnormalities using the National Cancer Institute Common Terminology Criteria for Adverse Events category and worst grade.

### Statistical analysis

The present study compared regorafenib combination therapy and monotherapy. Analyses were performed using the Statistical Package for the Social Science (IBM SPSS version 22.0; International Business Machines Corp, New York, USA). We compared the overall survival and progression-free survival using a stratified log-rank test and calculated HRs (with 95% CIs) using the Cox model, adjusting for baseline stratification factors. We calculated Kaplan-Meier survival estimates for each treatment group. We provide descriptive statistics and 95% CIs for overall survival and progression-free survival analyses.

The study was designed to confirm our hypothesis that combination therapy would extend median PFS and OS. The PFS in the study group was expected to be 3.5 months compared with 1.7 month based on the results of a previous study. A two-sided log rank test with an overall sample size of 68 subjects (34 in the control group and 34 in the treatment group) achieves 80.4% power at a 0.050% significance level to detect a hazard ratio of 00.4857.

### Role of the funding source

The present study received little funding. The present study was a retrospective, double arm cohort study for the mCRC patients treated in single institute. Collection of data, medical documents, laboratory data, and statistical analyses were conducted by the authors and the Biostatistics Task Force of Taichung Veterans General Hospital, who were responsible for the study results.

## Results

The baseline characteristics of both groups were not significantly different. In the combination treatment group, regorafenib was combined with FOLFIRI in 4 (11.8%) patients, Ufur/Xeloda in 7 (20.6%) patients, FOLFOX in 2 (5.8%) patients, Irino in 19 (55.9%) patients, and oxaliplatin in 2 (5.8%) patients. There were 2 patients who received a combination of regorafenib with oxaliplatin, and these individuals had undergone FOLFOX for second line therapy without discomfort. Oxaliplatin was added to regorafenib. By dose-escalating protocol, initial dosage was 80 mg per day in 29 (85.3%) patients in the combination group and 24 (88.9%) patients in the single use group. A total of 160 mg per day in 3 (8.8%) patients in the combination group and 3 (11.1%) patients in the single use group. Regorafenib was used as a second line treatment after metastasis was confirmed in 10(30.3%) patients in the combination group and 8(29.6%) patients in the single use group. In third line treatment in 18(54.5%) patients in the combination group and 14(51.9%) patients in the single use group. Fourth line treatment: 5(15.2%) vs 5 (18.5%). Before regorafenib, 20(58.8%) patients had received Avastin, 3(8.8%)patients had received cetuximab, and 11(32.4%) patients received both of treatments in the combination group. Avastin: 17(63.3%); cetuximab: 6(22.2%); Both: 4(14.8%) in the single use group.

Median follow up time was 10.4 months in the combination group and 6.1 months in the single use group. After regorafenib treatment with a dose-escalation protocol, there were 2(6.9%) patients with increased dose in the combined use group and 2(8.3%) patients in the single use group. A total of 4(13.8%) patients showed a decreased dose in the combined use group and 3(12.5%) patients in the single use group. The final regorafenib daily dosages were 40 mg in 4/34(11.8%), 80 mg in 27/34(79.4%), 120 mg 1/34(2.9%), 160 mg in 2/34(5.9%) in combined use group and 3/27(11.1%), 19/27(70.4%), 2/27(7.4%), and 3/27(11.1%) in the single use group. The dosage of regorafenib was not different between combination use and single use. Median therapeutic duration was 4.5 months(2.8–6.8) in the combination group and 2.9 months(2.0–4.0) in the single use group(*p* = 0.037). Disease control rate after three months treatment was 47.1%(16/34) in the combination group and 11.1%(3/27) in the single use group. Ending of regorafenib treatment was due to disease progression: 70.6%(24/34) in the combination group and 70.4%(19/27) in the single use group; or adverse effects: 23.5%(8/34) vs 25.9%(7/27). The adverse effects of combination use did not increase the risk of treatment failure(Table 2).

**Table 2:** Results of the present study.

	Single use (n = 27)		Combine (n = 34)		*p* value
	n	%	n	%	
**Final dosage (mg)**					0.734
40	3	(11.1%)	4	(11.8%)	
80	19	(70.4%)	27	(79.4%)	
120	2	(7.4%)	1	(2.9%)	
160	3	(11.1%)	2	(5.9%)	
**Response after 3 months treatment**					0.006**
Progress Disease (PD)	24	(88.9%)	18	(52.9%)	
Stable disease (SD)	3	(11.1%)	16	(47.1%)	
**Reasons to stop regorafenib**					0.914
Progress Disease (PD)	19	(70.4%)	24	(70.6%)	
Adverse events (AE)	7	(25.9%)	8	(23.5%)	
Regorafenib was ongoing	1	(3.7%)	2	(5.9%)	
**Duration of regorafenib**	2.9	(2.0, 4.0)	4.5	(2.8, 6.8)	0.037*
**Follow-up time**†					
OS	6.1	(5.3, 10.5)	10.4	(5.7, 13.1)	0.058
PFS	2.5	(1.6, 3.6)	4.0	(2.5, 5.9)	0.014*

Chi-square test.

†Mann-Whitney U test, Median (IQR)

*p<0.05

**p<0.01

**Fig 3:**
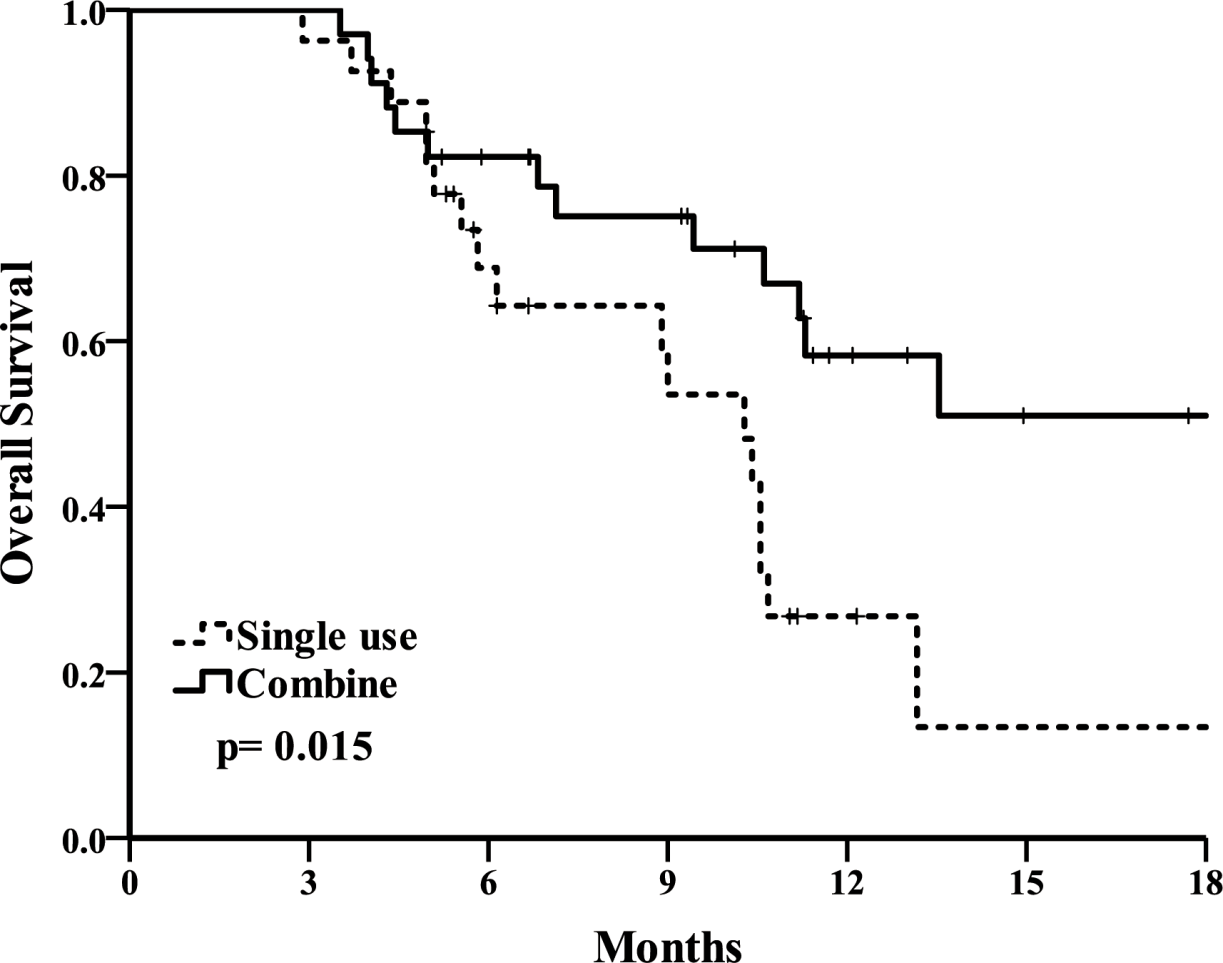
Overall survival Kaplan-Meier analysis of combination and single use group.

**Fig 4:**
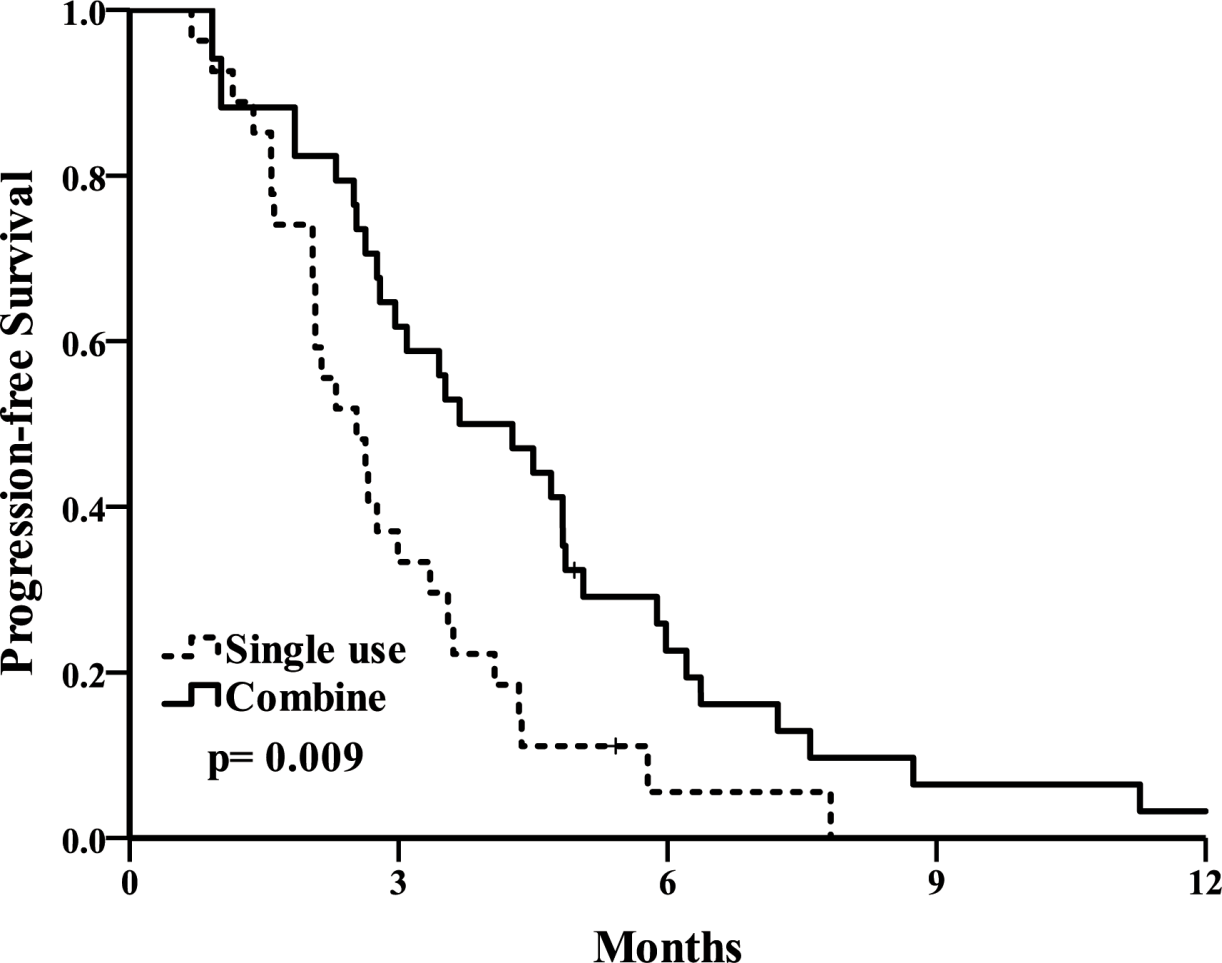
Progression-free survival Kaplan-Meier analysis of combination and single use group.

The primary end point of overall survival(OS). The OS curve is shown in Fig 3. Median OS was 20.9(10.1–31.7) months in the combination group and 10.3(8.3–12.3) months in the single use group (*p* = 0.015). A second end point of progression-free survival(PFS) (Fig 4) was 3.7(2.2–5.2) months in the combination group and 2.5(1.7–3.4) months in the single use group (*p* = 0.009). A total of 58.3% patients remained alive at the 1-year follow up in the combination group and 26.8% patients in the single use group. The combination group had longer overall survival and progression-free survival.

Overall, there were 27 patients (79.4%) suffered from any grade of adverse effect in combination group and 18 patients (66.6%) in single use group (Table 3). Most frequently seem were hand food skin reaction (HFSR), gastrointestinal discomfort, and fatigue. Adverse effects predominantly noted in combination group were mucositis (14.7% vs 3.7%), diarrhea (11.8% vs 3.7%), neutropenia (11.8% vs 3.7%), and thrombocytopenia(2.9% vs 0%).

**Table 3:** Adverse effects occurring after treatment.

	Single use (n = 27)		Combine (n = 34)		*p* value
	n	%	n	%	
**Any events**	18	(66.6%)	27	(79.4%)	0.406
**HFSR**	12	(44.4%)	16	(47.1%)	1.000
**headache**	1	(3.7%)	1	(2.9%)	1.000
**mucositis**	1	(3.7%)	5	(14.7%)	0.317
**N/V**	6	(22.2%)	7	(20.6%)	1.000
**Diarrhea**	1	(3.7%)	4	(11.8%)	0.371
**Hypertension**	1	(3.7%)	0	(0.0%)	0.443
**Fatigue**	7	(25.9%)	6	(17.6%)	0.639
**Discomfort**	2	(7.4%)	0	(0.0%)	0.374
**Liver function elevation**	2	(7.4%)	3	(8.8%)	1.000
**Thrombocytopenia**	0	(0.0%)	2	(5.9%)	0.498
**Hair loss**	0	(0.0%)	2	(5.9%)	0.498
**Neutropinia**	1	(3.7%)	4	(11.8%)	0.371

Chi-square test. *p<0.05, **p<0.01

## Discussion

The optimal dosage for regorafenib is not known. The suggested dosage was 160mg per day for 3 weeks and rest for 1 week according to the best drug concentration recommended in a previous study[23]. However, drug compliance was not good and most patients needed adjustments. The minimal daily dose of regorafenib allowed was 80mg[24]. This dose was selected as no data on antitumor activity was generated with lower concentrationsin a previous study[25]. Thus, certain physicians started regorafenib at 80 mg daily for the first week, escalated the dosage to 120 mg daily for the second week if no significant side effect was observed, and subsequently escalated the dosage again to 160 mg daily, for the third week, then scheduled a break for the fourth week[16]. At our institute, most physicians initiated regorafenib at the 80-mg daily dose. Dosage up-regulation was determined at the physicians’ discretion. Most patients remained at 80 mg daily at our institute.

Another real-life regorafenib experience was shared by Ka-On Lam et al., who recruited 45 patients in three institutes in Hong Kong[26]. A total of 25 patients were treated at a lower dose, and 20 patients were started at a full dose. Median overall survival time was not inferior in low dose regorafenib (51.3 weeks vs 27.4 weeks, p = 0.449) nor was median progression-free survival time (18.7 weeks vs 13.4 weeks, *p* = 0.458). The adverse effects of a full dose of regorafenib made this dosage not practical in every case. Hence, low dose initiation was relatively feasible in clinical practice and might confer the same effect.

There was a Phase IB study by B. Schiltheis that analyzed the combination of regorafenib with FOLFOX or FOLFIRI in first or second line chemotherapy[27]. The study recruited 45 patients using combination therapy with regorafenib 160 mg daily and standard FOLFOX or FOLFIRI. Median PFS was 126 days. Similar PFS between first line and second therapy either combined with FOLFOX or FOLFIRI was noted. Adverse effects were acceptable. However, combination use was not suggested due to more adverse effects and non-superior outcomes to chemotherapy alone.

Regorafenib combination use was reported by Wang et al[28]. Regorafenib combined with FOLFIRI was used in a metastatic colorectal cancer patient in fourth line therapy after FOLFIRI, FOLFOX, cetuximab, and bevacizumab were used. The dosage of regorafenib was 160mg daily and FOLFIRI was initiated from 180 mg/m^2^ for irinotecan with subsequent up regulation to 290 mg/m^2^. A partial response was achieved and PFS was longer than 6 months. Currently, there are no cases concerning regorafenib combination use in third line chemotherapy. The optimal regimens for regorafenib combination therapy are also not known. We used sub group different combination therapy and observed overall survival time. The results are shown in Table 4.

**Table 4:** Overall survival time between different chemotherapy treatments in the combination group.

	n	Mean	Std	Median
XeLIRI or FOLFIRI	5	10.6	5.6	10.6
Ufur or Xeloda	7	11.0	3.8	11.3
FOLFOX	1	21.2		
Irino	19	10.0	5.6	9.3
Oxaliplatin	2	8.2	5.5	

The optimal dosage for combination therapy was not defined. Physicians administered dosages according to a previous study. There were no severe adverse effects noted in the present study after these regimens were combined with regorafenib. The outcome of regorafenib combination use was superior to that of single use.

## Conclusion

Regorafenib combination use with conventional chemotherapy, either single regimen 5-FU, irinotecan, oxaliplatin, or combination regimen FOLFIRI/ FOLFOX, brought superior survival benefit than single use. Side effects were tolerable without decreasing drug compliance. Optimal regimen and dosage of the combination treatment are pending future studies.

## Supporting information

S1 File The file contains the raw data underlying the present study. (XLSX)
